# Excessive expression of miR-1a by statin causes skeletal injury through targeting mitogen-activated protein kinase kinase kinase 1

**DOI:** 10.18632/aging.202839

**Published:** 2021-04-16

**Authors:** Chang-Ning Fu, Jia-Wen Song, Zhi-Peng Song, Qian-Wen Wang, Wen-Wu Bai, Tao Guo, Peng Li, Chao Liu, Shuang-Xi Wang, Bo Dong

**Affiliations:** 1 The Key Laboratory of Cardiovascular Remodeling and Function Research, Chinese Ministry of Education, Chinese National Health Commission and Chinese Academy of Medical Sciences, The State and Shandong Province Joint Key Laboratory of Translational Cardiovascular Medicine, Department of Cardiology, Qilu Hospital, Cheeloo College of Medicine, Shandong University, Jinan, China; 2 Department of Cardiology, Shandong Provincial Hospital, Shandong University, Jinan, China; 3 Henan International Joint Laboratory of Cardiovascular Remodeling and Drug Intervention, School of Pharmacy, Xinxiang Medical University, Xinxiang, China; 4 Hubei Key Laboratory of Diabetes and Angiopathy, Hubei University of Science and Technology, Xianning, China

**Keywords:** statin, microRNA-1a, mitogen-activated protein kinase kinase kinase 1, apoptosis, myopathy

## Abstract

Backgrounds: A major side effect of statin, a widely used drug to treat hyperlipidemia, is skeletal myopathy through cell apoptosis. The aim of this study is to investigate the roles of microRNA in statin-induced injury.

Methods: Apolipoprotein E knockout (ApoE-/-) mice were administered with simvastatin (20 mg/kg/day) for 8 weeks. Exercise capacity was evaluated by hanging grid test, forelimb grip strength, and running tolerance test.

Results: In cultured skeletal muscle cells, statin increased the levels of miR-1a but decreased the levels of mitogen-activated protein kinase kinase kinase 1 (MAP3K1) in a time or dose dependent manner. Both computational target-scan analysis and luciferase gene reporter assay indicated that MAP3K1 is the target gene of miR-1a. Statin induced cell apoptosis of skeletal muscle cells, but abolished by downregulating of miR-1a or upregulation of MAP3K1. Further, the effects of miR-1a inhibition on statin-induced cell apoptosis were ablated by MAP3K1 siRNA. In ApoE-/- mice, statin induced cell apoptosis of skeletal muscle cells and decreased exercise capacity in mice infected with vector, but not in mice with lentivirus-mediated miR-1a gene silence.

Conclusion: Statin causes skeletal injury through induction of miR-1a excessive expression to decrease MAP3K1 gene expression.

## INTRODUCTION

Statins are widely used to treat hyperlipidemia and lower cardiovascular disease risks because they inhibit HMG-CoA reductase [1]. Statin also produces multiple side effects, such as muscle symptoms, diabetes mellitus, abdominal aortic aneurysm, and central nervous system complaints, limiting the applications in some patients [2, 3]. As for muscle symptoms, it includes statin-associated myalgia, rhabdomyolysis and necrotizing autoimmune myopathy, etc, in which the apoptosis of skeletal muscle cell plays critical roles [4, 5]. The molecular mechanism of skeletal muscle cell apoptosis needs further investigation.

MicroRNAs are approximately 20-nucleotide, single-stranded RNA molecules by targeting the 3′ untranslated regions (3′-UTR) of specific mRNAs through partial complementarity [6]. In this way, miRNAs regulate gene expressions through inhibition of translation or transcript degradation. Interestingly, we have reported that statin inhibits miR-133a, specifically and highly expressed in skeletal and cardiac muscles, and ectopically expressed in endothelial cells under pathophysiological conditions [7, 8]. This demonstrates that statin is able to regulate myo-miRNA gene expressions.

As previously reported, miR-103/107, miR-208 and miR-21 are biomarkers of skeletal muscle toxicity [9], which are involved in myocyte apoptosis [10, 11], indicating several miRNAs are critical in the regulation of muscle cell function and fate determination. Some studies have demonstrated that overexpression of muscle-specific microRNAs, such as miR-1, leads to cell apoptosis and regulates skeletal muscle development [12–14]. Whether miR-1a is related to statin-induced skeletal injury remains unclear.

Based on aforementioned observations, we tested the hypothesis that statin causes skeletal injury through miR-1a-mediated of cell apoptosis. Here we reported that mitogen-activated protein kinase kinase kinase 1 (MAP3K1) is a target of miR-1a and simvastatin decreases MAP3K1 gene expression to induce skeletal muscle cell apoptosis to cause skeletal muscle injury by enhancement of miR-1a *in vitro* and *in vivo*. In perspectives, normalization of miR-1a or MAP3K1 dysregulation should be taken into consideration to prevent statin-induced myopathy clinically.

## MATERIALS AND METHODS

An expanded Materials and Methods section is available in the Supplementary Materials.

### Animals and experimental protocols

Apolipoprotein E knockout (*ApoE^-/-^*) mice, 6-8 weeks of age, were purchased from Beijing Huafukang Animal Experimental Center (Beijing, China). The gender is mixed, and we tried to have 50% of each gender in each group. All animals were housed in temperature-controlled cages with a 12-hour light-dark cycle. Simvastatin were given to mice by gavage at a dose of 20 mg/kg/day for 8 weeks to induce skeletal injury as reported previously [15, 16]. This study was carried out in accordance with the ethical standards laid down in the 1964 Declaration of Helsinki and its later amendments. The animal protocol was reviewed and approved by the Animal Care and Use Committee of Shandong University Qilu Hospital.

### Cell culture of skeletal muscle cells

As described previously [17], gastrocnemius muscle from 10 days old suckling mice (C57BL/6) was digested by collagenase II and trypsin at 37° C. After centrifugation, cells were suspended in DMEM medium containing 10% fetal bovine serum. Skeletal muscle satellite cells were isolated by using the difference of the adhesion speed with fibroblasts. 1. 2 × 10^6^ cells were seeded in each well of 6-well plate. When the cell density reached 80-90%, medium was replaced with DMEM medium containing 2% horse serum, and the medium was changed every day. After 3 days, cells began to differentiate. After 5 days, 90% of satellite cells differentiated into mature skeletal muscle cells with the formation of myotubes and MHC expression (Supplementary Figure 1).

### RNA extraction and real-time PCR

Extract total RNA from skeletal muscle cells or tissues using TRIzol Reagent according to manufacturer's instructions [18, 19].

### Luciferase reporter assay

As described previously [7, 20], plasmid constructs (MAP3K1-UTR or MT-MAP3K1-UTR) were co-transfected in HEK293 cells with the pCMV β-gal plasmid and 50 nM each of chemically synthesized miR-1a or negative control oligonucleotides by using lipofectamine 2000. Cells were harvested 48 hours after transfection, and luciferase and β-galactosidase activities were measured.

### Determination of exercise capacity

The exercise capacity was determined using forelimb grip strength test, hanging grid test, and exhaustive running test as previously reported [16].

### Statistical analysis

Data are reported as mean ± SEM. A one-way ANOVA followed by Bonferroni correction was used for multiple comparisons. Two-sided *P*-value <0.05 was considered as significant.

## RESULTS

### Statin increases the expression of miR-1a in skeletal muscle cells

**Figure 1:**
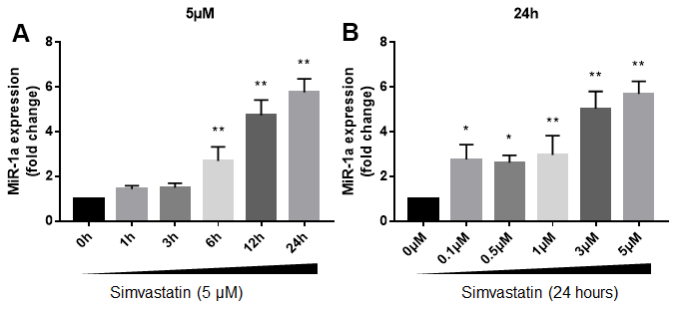
**Statin induces excessive expression of miR-1a in skeletal muscle cells.** (**A**) Cultured skeletal muscle cells were treated with simvastatin (5 μM) as indicated time points. The expressions of miR-1a was assayed using quantitative real-time PCR. N = 5 per group. ^**^P < 0.01 vs. control group (0 h). (**B**) Cultured skeletal muscle cells were treated with simvastatin (24 hours) as indicated concentration points. The expressions of miR-1a was assayed using quantitative real-time PCR. N = 5 per group. ^*^P < 0.05 or ^**^P < 0.01 vs. control group (0 μM). A one-way ANOVA followed by Tukey *post-hoc* tests was used to determine *P* value in (**A**, **B**).

We firstly investigated the effects of statin in miR-1a expression in cultured skeletal muscle cells. Cultured skeletal muscle cells (originally differentiated from gastrocnemius skeletal muscle satellite cells isolated from suckling mice) were treated with varying concentrations of simvastatin from 1 to 24 h. The expressional level of miR-1a in skeletal muscle cells gradually increased beginning 6 h after incubation with 5 μM of simvastatin and continued to increase at 12h and 24h (Figure 1A). Next, we examined the dose-dependent effects of simvastatin on miR-1a expression (Figure 1B). Simvastatin increased miR-1a expression at a concentration of 0.5 μM. Increasing concentrations of simvastatin (1-5 μM) further enhanced miR-1a expression. These data indicate that statin induces excessive expression of miR-1a in skeletal muscle cells.

### Statin induces skeletal muscle cell apoptosis through miR-1a

**Figure 2:**
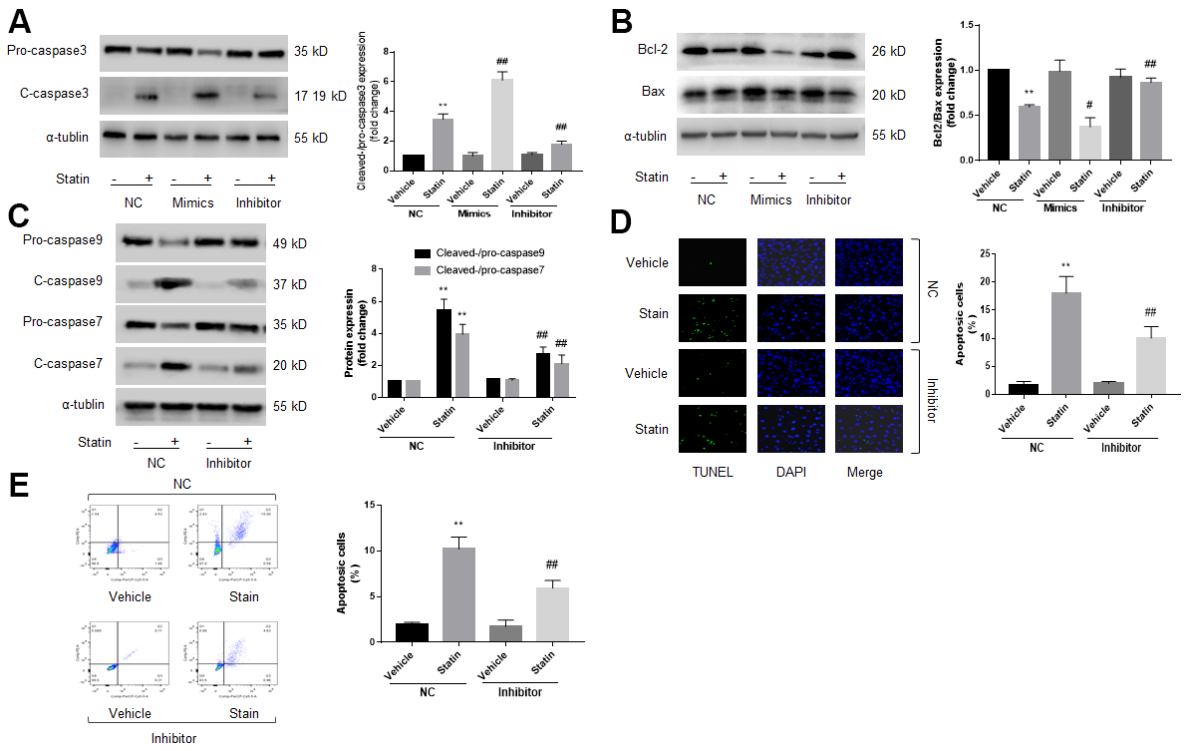
**Statin induces cell apoptosis via upregulation of miR-1a in skeletal muscle cells.** (**A**–**C**) Cultured skeletal muscle cells were transfected with miR-1a mimics and inhibitors for 48 hours followed by statin treatment for 24 hours. Cells were subjected to detect the protein levels of pro-/cleaved-caspase3 in (**A**) bcl-2 and bax in (**B**) and pro-/cleaved-caspase7, 9 in (**C**) by western blot. N is 5 in each group. ^**^P<0.01 vs. NC alone. ^#^*P*<0.05 or ^##^*P*<0.01 vs. statin alone. (**D**, **E**) Cultured skeletal muscle cells were transfected with miR-1a inhibitor for 48 hours followed by statin treatment for 24 hours. Cell apoptosis was determined by TUNEL method in (**D**) and flow cytometry in (**E**). N is 5 in each group. ^**^P<0.01 vs. NC alone. ^##^*P*<0.01 vs. statin alone. A one-way ANOVA followed by Tukey *post-hoc* tests was used to determine *P* value in (**A**–**E**).

Previous studies had reported that miR-1a mediates cell apoptosis [12, 13]. Thus, we examined the role of miR-1a in statin-induced cells apoptosis by transfecting miR-1a inhibitor or mimics into skeletal muscle cells, which was specially changed miR-1a expression (Supplementary Figure 2A), indicating the on-target effect. Protein levels of pro-/cleaved-caspase3, 7, 9, bcl-2, and bax, were assayed by western blot to represent cell apoptosis. As shown in Figure 2A–2C, simvastatin increased the ratio of cleaved-caspase3, 7, 9 to pro- caspase3, 7, 9 and decreased the ratio of bcl-2 to bax. As expected, these effects of statin were inhibited by miR-1a inhibitor but exacerbated by miR-1a mimics.

Cell apoptosis was assayed by using TUNEL and flow cytometry methods. As presented in Figure 2D, 2E, similar to apoptosis-related proteins, statin significantly induced cell apoptosis of skeletal muscle cells, which were bypassed by miR-1a inhibitor. Taking these data together, it suggests that satin induces skeletal muscle cell apoptosis through miR-1a activation. Cell apoptosis was further confirmed by measuring cell viability using CCK8 method as described previously [21]. Statin significantly decreased the number of viable cells in cells transfected with miR-1a inhibitor negative control, but not in cells transfected with miR-1a inhibitor (Supplementary Figure 2B).

### MAP3K1 is a target gene of miR-1a

**Figure 3:**
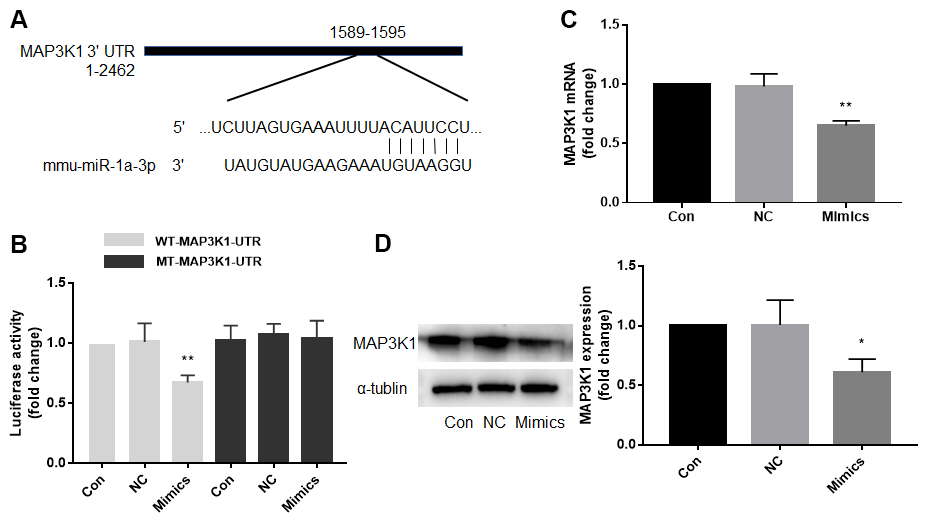
**Mitogen-activated protein kinase kinase kinase 1 (MAP3K1) is a target gene of miR-1a.** (**A**) Computational target-scan analysis showed that miR-1a is able to bind the highly conserved target site (1589-1595, 5′-ACAUUCC-3′) in the 3′-UTR of MAP3K1 mRNA. (**B**) Plasmid of luciferase reporter construction containing 3′-UTR of MAP3K1 mRNA (WT-MAP3K1-UTR) or mutant of MAP3K1-UTR (MT-MAP3K1-UTR) was co-transfected with miR-1a negative control (NC) or mimics in HEK293 cells. The luciferase activities in total cell lysates were assayed. N is 5 in each group. ^**^P<0.01 vs. NC. (**C**, **D**) Cultured skeletal muscle cells were transfected with miR-1a NC and mimics for 48 hours. The mRNA level of MAP3K1 in (**C**) and protein level in (**D**) were analyzed using real-time PCR and western blot, respectively. N is 5 in each group. ^*^P < 0.05 or ^**^P < 0.01 vs. NC. A one-way ANOVA followed by Tukey *post-hoc* tests was used to determine *P* value in (**B**–**D**).

Computational target-scan analysis (Figure 3A) showed that miR-1a potentially binds to the highly conserved target site (1589-1595, 5′-ACAUUCC-3′) in the 3′-UTR of MAP3K1 mRNA. This suggested that miR-1a may inhibit the expression of MAP3K1 through post-transcriptional regulation. To examine if miR-1a can repress MAP3K1 gene expression through direct 3′-UTR interaction, we cloned MAP3K1 3′-UTR luciferase reporter plasmid and performed reporter analysis in HEK293 cells. As shown in Figure 3B, co-transfection of miR-1a with plasmid of MAP3K1 3′-UTR reporter resulted in a significant inhibition of luciferase activity. Further, miR-1a failed to suppress the activity of MAP3K1 3′-UTR reporter with a mutated miR-1a seed sequence. These data indicated that MAP3K1 mRNA is a direct target of miR-1a. Moreover, we measured the effects of overexpression of miR-1a on MAP3K1 mRNA using real-time PCR (Figure 3C) and protein using Western blot (Figure 3D). Overexpression of miR-1a significantly decreased both mRNA and protein levels of MAP3K1, compared to negative control, further supporting the notion that miR-1a functions as a regulator of MAP3K1 gene expression.

### Statin decreases the protein expression of MAP3K1 in cells

Knowing MAP3K1 is a target gene of miR-1a and statin increases miR-1a expression in skeletal muscle cells, we thought that statin reduces MAP3K1 levels in skeletal muscle cells. As expected, statin decreased the protein expression of MAP3K1 in cultured skeletal muscle cells, also in a dose or time dependent manner (Supplementary Figure 3A, 3B).

### Statin induces skeletal muscle cell apoptosis via MAP3K1 deficiency

**Figure 4:**
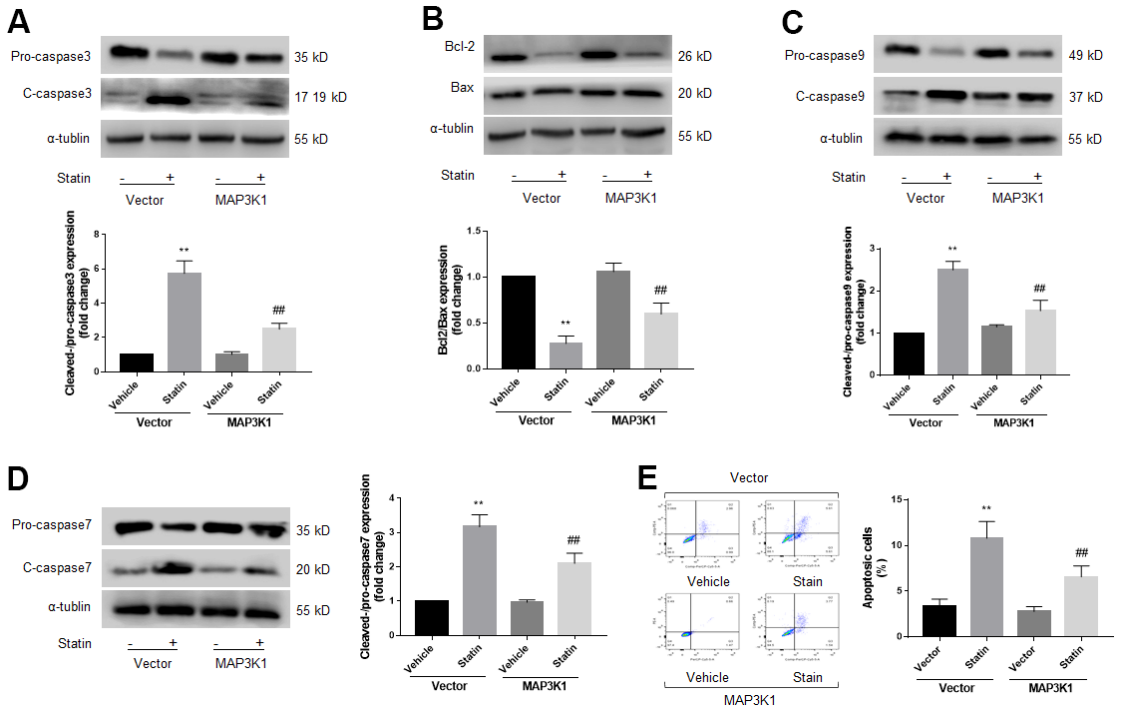
**Statin induces cell apoptosis of skeletal muscle cells via downregulation of MAP3K1.** Cultured skeletal muscle cells were transfected with plasmids MAP3K1 cDNA for 48 hours followed by statin treatment for 24 hours. Cells were subjected to detect the protein levels of pro-/cleaved-caspase3 in (**A**) bcl-2 and bax in (**B**) pro-/cleaved-caspase9 in (**C**) and pro-/cleaved-caspase7 in (**D**) by western blot. Cell apoptosis was determined by flow cytometry in (**E**). N is 5 in each group. ^**^P<0.01 vs. vector alone. ^##^*P*<0.01 vs. statin plus vector. A one-way ANOVA followed by Tukey *post-hoc* tests was used to determine *P* value in (**A**–**E**).

MAP3K1 is a key factor to control cell apoptosis [22]. Therefore, we determined whether MAP3K1 plays roles in statin-induced skeletal muscle cell apoptosis. As shown in Figure 4A–4D, overexpression of MAP3K1 dramatically inhibited the effects of statin on caspase3, 7, 9, bcl-2, and bax in skeletal muscle cells. Consistently, overexpression of MAP3K1 also abrogated statin-induced cell apoptosis of skeletal muscle cells as determined by flow cytometry (Figure 4E). As shown by the result of CCK8, overexpression of MAP3K1 increased the number of viable cells, which was excessively reduced by statin stimulation (Supplementary Figure 3D).

### Statin induces cell apoptosis through the miR-1a-MAP3K1 pathway

**Figure 5:**
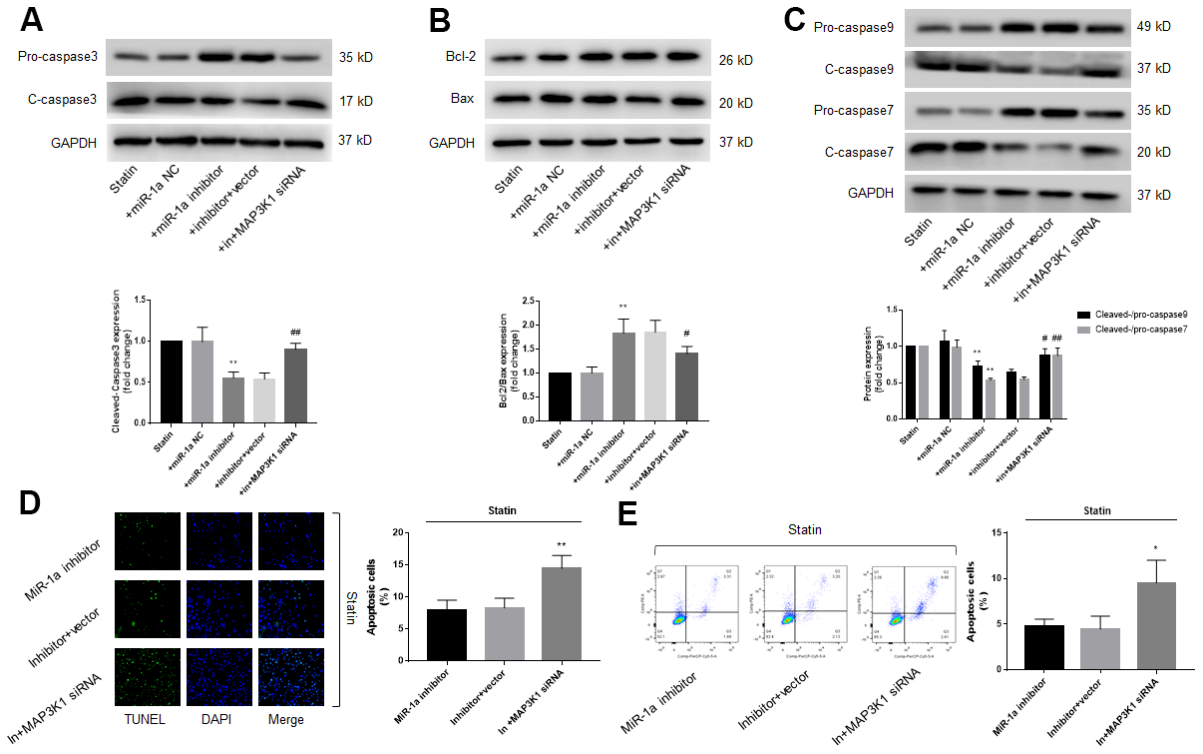
**The miR-1a-MAP3K1 pathway mediates statin-induced cell apoptosis in skeletal muscle cells.** Cultured skeletal muscle cells were transfected with miR-1a inhibitor and/or MAP3K1 siRNA for 48 hours followed by statin treatment for 24 hours. (**A**–**C**) Cells were subjected to detect the protein levels of pro-/cleaved-caspase3 in (**A**) bcl-2 and bax in (**B**) and pro-/cleaved-caspase7, 9 in (**C**) by western blot. N is 5 in each group. ^**^P<0.01 vs. statin alone. ^#^*P*<0.05 or ^##^*P*<0.01 vs. statin plus miR-1a inhibitor. (**D**, **E**) Cell apoptosis was determined by TUNEL in (**D**) and flow cytometry in (**E**). ^*^P < 0.05 or ^**^P < 0.01 vs. statin plus miR-1a inhibitor. A one-way ANOVA followed by Tukey *post-hoc* tests was used to determine *P* value in (**A**–**E**).

Since either miR-1a inhibition or MAP3K1 overexpression prevents statin-induced skeletal muscle cell apoptosis, we thought that MAP3K1 may function as a downstream mediator of miR-1a in regulation of cell apoptosis. To this viewpoint, we co-transfected cells with miR-1a inhibitor and MAP3K1 siRNA. As shown in Figure 5A–5C, though statin-induced alterations of caspase3, 7, 9, bcl-2 and bax proteins were abolished by miR-1a inhibitor, miR-1a inhibitor had no effects in statin-treated cells if transfected with MAP3K1 siRNA. Both TUNEL assay and flow cytometry indicated that the inhibitory effects of miR-1a inhibitor on statin-induced apoptosis were completely prevented by MAP3K1 siRNA (Figure 5D, 5E). CCK8 showed consistent result (Supplementary Figure 4A, 4B). Together, these data suggest that statin induces cell apoptosis through the miR-1a-MAP3K1 pathway in skeletal muscle cells.

### Inhibition of miR-1a prevents statin-induced muscle injury in mice

**Figure 6:**
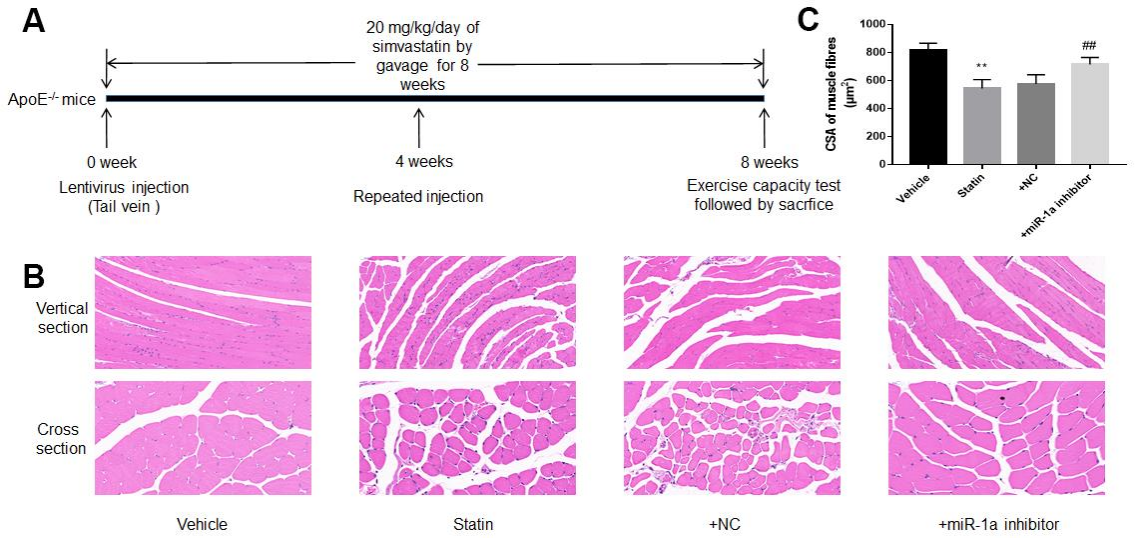
**Downregulation of miR-1a prevents statin-induced morphological deterioration of gastrocnemius in *ApoE^-/-^* mice.** (**A**) The protocol for animal experiments was shown. (**B**) The morphology of gastrocnemius muscle was observed by HE staining. (**C**) Quantitative analysis of fibre cross-sectional area (CSA) in gastrocnemius muscle from mice was performed. N is 10-15 in each group. ^**^P < 0.01 vs. vehicle group. ^##^*P*<0.01 vs. statin alone. A one-way ANOVA followed by Tukey *post-hoc* tests was used to determine *P* value in (**C**).

**Figure 7:**
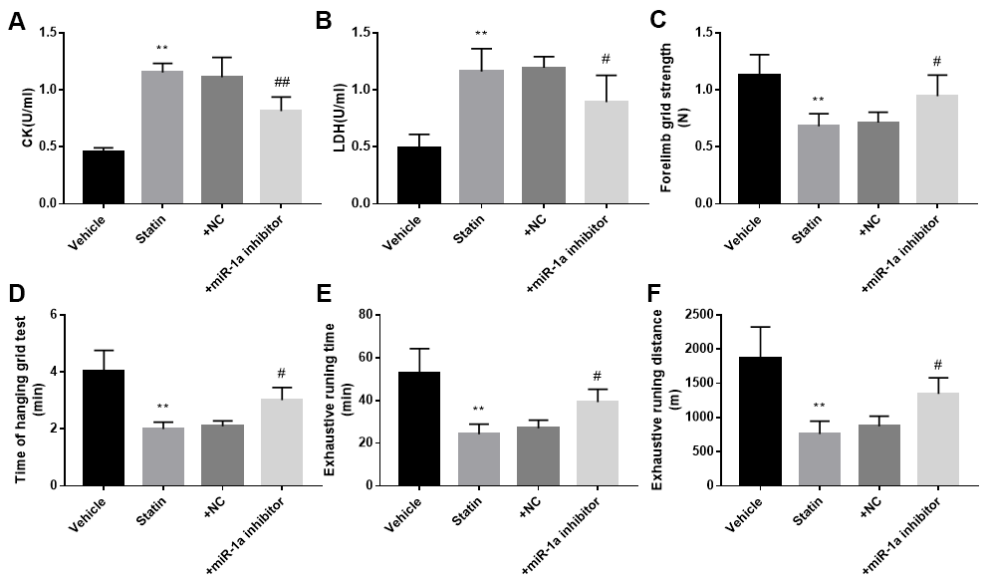
**Inhibition of miR-1a improves the exercise ability in mice treated with statin.** The protocol for animal experiments was shown in Figure 6A. (**A**, **B**): The levels of creatine kinase (CK) and lactate dehydrogenase (LDH) in plasma from mice were assayed. (**C**–**F**) The exercise capacity was determined, and the forelimb grip strength in (**C**) hanging grid time in (**D**) exhaustive running time in (**E**) and distance of exhaustive running in (**F**) were calculated. N is 10-15 in each group. ^**^P < 0.01 vs. PBS group. ^#^*P*<0.05 or ^##^*P*<0.01 vs. statin alone. A one-way ANOVA followed by Tukey *post-hoc* tests was used to determine *P* value in (**A**–**F**).

We next investigated the roles of miR-1a in statin-induced skeletal injury *in vivo*. *ApoE^-/-^* mice, with the background of natural hyperlipidemia [23, 24], was selected in this study. The protocol for animal experiment was shown in Figure 6A. HE staining of gastrocnemius muscle revealed marked variations in skeletal muscle fibre size and arrangement, as well as decreased fibre cross-sectional area (CSA) in statin administration group. While, lentivirus-mediated miR-1a inhibition abrogated statin-induced fibre disorganization and CSA decrease (Figure 6B, 6C). Also, the indexes of muscle damage including CK and LDH, were normalized by miR-1a inhibition in statin-treated mice, compared to mice infected with lentivirus vector (Figure 7A, 7B). These data imply that inhibition of miR-1a improves statin-induced muscle damage *in vivo*.

### miR-1a inhibition improves statin-impaired exercise capacity in mice

Statin-induced skeletal injury was further assessed using exercise capacity by calculating forelimb grip strength test, hanging grid test, and exhaustive running test as reported previously [16]. As presented in Figure 7C–7F, statin administration reduced the exercise capacity in *ApoE^-/-^* mice infected with lentivirus vector, but not in *ApoE^-/-^* mice infected lentivirus expressing miR-1a inhibitor, further supporting that statin induces skeletal myopathy through induction of miR-1a in mice.

### Inhibition of miR-1a prevents statin-induced skeletal muscle cell apoptosis in *ApoE^-/-^* mice

**Figure 8:**
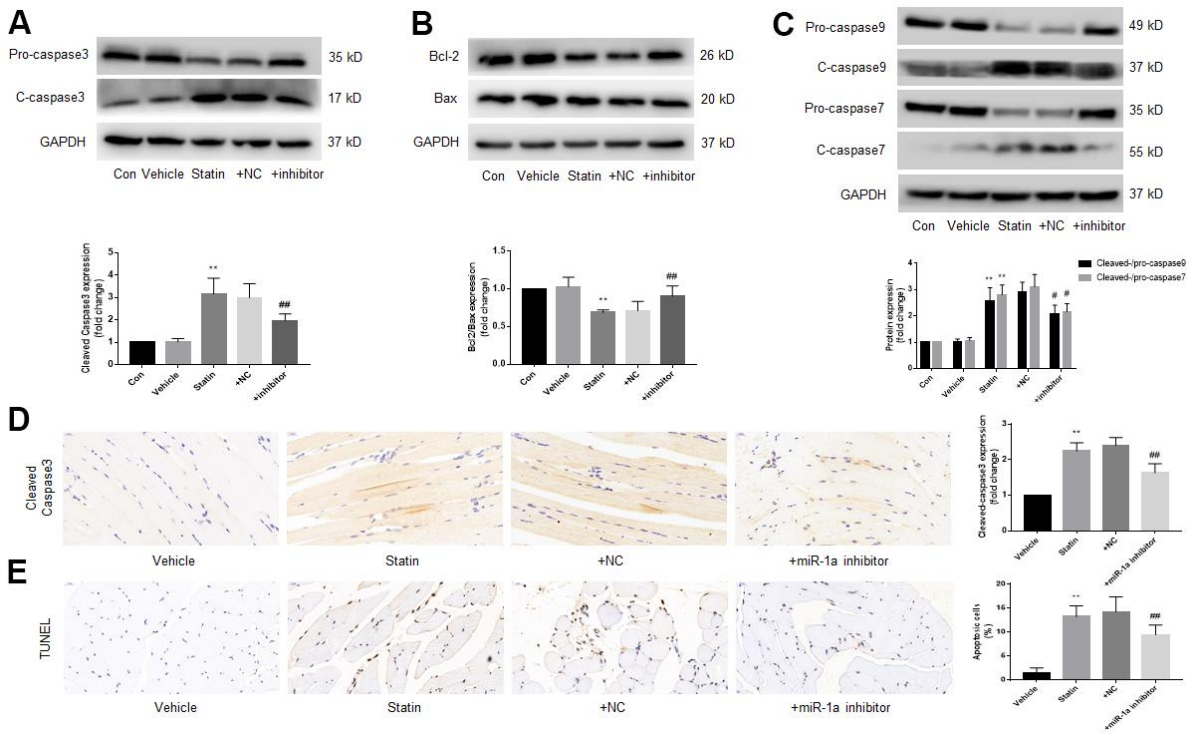
**Downregulation of miR-1a expression inhibits the apoptosis of gastrocnemius muscle cells induced by statin *in vivo*.** The protocol for animal experiments was shown in Figure 6A. (**A**–**C**) Expressional levels of pro-/cleaved-caspase3, 7, 9, bcl-2 and bax in gastrocnemius were assayed by western blot. (**D**) Cleaved-caspase3 in gastrocnemius were assayed by IHC. (**E**) Cell apoptosis in gastrocnemius was determined by TUNEL method. N is 10-15 in each group. ^**^P < 0.01 vs. vehicle group. ^#^*P*<0.05 or ^##^*P*<0.01 vs. statin alone. A one-way ANOVA followed by Tukey *post-hoc* tests was used to determine *P* value in (**A**–**E**).

Finally, we examined the effects of miR-1a inhibition on statin-induced cell apoptosis *in vivo*. As indicated in Supplementary Figure 5A, 5B, simvastatin upregulated miR-1a gene expression but downregulated MAP3K1 protein expression, which were reversed by lentivirus-mediated gene silence of miR-1a. Further, statin increased the ratio of cleaved-caspase3, 7, 9 to pro- caspase3, 7, 9, decreased the ratio of bcl-2 to bax (Figure 8A–8C), increased the expression of cleaved-caspase3 (Figure 8D) and induced cells apoptosis in gastrocnemius (Figure 8E). Importantly, all of these phenotypes induced by simvastatin were prevented by miR-1a downregulation.

### Body weight, food intake, and serum cholesterol content of mice during the experimental cycle

Relevant data are presented in Supplementary Tables 1–3.

## DISCUSSION

In this study, we firstly demonstrated that statin induced excessive expression of miR-1a to cause skeletal injury *in vivo*. Mechanically, miR-1a represses MAP3K1 gene expression by targeting of the 3'-UTR of mRNA to promote skeletal muscle cell apoptosis. To the best of our knowledge, this study is firstly to report that statin via induction of miR-1a decreases MAP3K1 to induce skeletal myopathy.

The major discovery of the study is that MAP3K1 is a target of miR-1a in skeletal muscle cells. It has been reported that miR-1a plays an important role in multiple cellular functions, such as differentiation of chondrocytes and skeletal muscle satellite cells [25, 26], maintaining normal rhythm of cardiomyocytes [27], and reinforcing the striated muscle phenotype [28]. In particular, miR-1a regulates the apoptotic process of many cells, such as prostate cancer cells [29], nerve cells [30], and cardiomyocytes [31]. In this study, we further exploited that MAP3K1 is a novel target of miR-1a in skeletal muscle cells. This was demonstrated by the following evidences: (1) miR-1a target site is found to be located in the 3’-UTR of MAP3K1 mRNA as indicated “5’-ACAUUCC-3’”; (2) Transfection of miR-1a decreased luciferase reporter activity in plasmid of MAP3K1 3′-UTR, but not in plasmid of MAP3K1 3′-UTR with the mutant of miR-1a target site; (3) MAP3K1 as a target of miR-1a was further supported in both cultured cells and *ApoE^-/-^* mice, in which loss-function of miR-1a increased MAP3K1 gene expression. This discovery not only uncovers a novel mechanism of MAP3K1 posttranslational gene regulation, but also broadens the biological functions of miR-1a.

In the present study, we further illustrated that statin induces miR-1a to cause skeletal muscle cell apoptosis and injury *in vitro* and *in vivo*. We transfected skeletal muscle cells with miR-1a mimics and inhibitor, respectively. From the results of apoptosis-related proteins, TUNEL assay and flow cytometry, it can be affirmatory that statin increases the apoptotic level of skeletal muscle cells, while the apoptotic level is further increased after overexpression of miR-1a, and is suppressed after inhibition of miR-1a. CCK8 results showed that statin reduced the number of viable cells, while inhibition of miR-1a increased the viable cell number, which indicated decreased cytotoxicity. *In vivo*, lentivirus-mediated inhibition of miR-1a similarly suppressed statin induced apoptosis in skeletal muscle, as indicated by apoptosis related-proteins and TUNEL.

MAP3K1 has both anti- and pro-apoptotic functions depending on the cell type and stimulus [22]. In order to determine whether MAP3K1 has anti-apoptotic or pro-apoptotic effects on statin-induced skeletal muscle cell injury, we overexpressed MAP3K1 in statin-treated skeletal muscle cells. According to the results reflected by western blot, flow cytometry and TUNEL assay, overexpression of MAP3K1 can improve the cell apoptosis. By inhibiting the expression of MAP3K1 through siRNA, the anti-apoptotic effect of miR-1a inhibition was prevented. This indicates that MAP3K1 of skeletal muscle cells has anti-apoptotic effect under the stimulation of statins. The miR-1a-MAP3K1 pathway, which is firstly reported by us, is involved in statin-induced skeletal muscle toxicity.

Traditionally, statin has been reported to prevent cardiovascular diseases [32], but produces some side effects including skeletal dysfunctions. Multiple mechanisms have been suggested contributing to statin toxicity, such as reduced sarcolemma or T-tubule cholesterol [33], reduction in CoQ10 [34], activation of the phosphoinositide 3 kinase pathway [2], and impaired mitochondrial function [35]. In this study, our data indicate that the detrimental effects of statin on skeletal function depend on miR-1a because lentivirus-mediated inhibition of miR-1a prevented statin-reduced exercise capacity in *ApoE^-/-^* mice. Thus, we reveal a uniform miR-1a-mediated mechanism of statin on impairing skeletal functions. Of note, how statin induces miR-1a expression in skeletal cells needs further investigations.

There are many miRs and proteins related to myoblast proliferation and differentiation, such as miR-133a, miR-206, SRF, mTOR/MyoD and MEF2, and we detected the expression of these miRs and proteins under statin stimulation. The results showed that, compared with the control group, the expression of miR-133a, SRF increased, while the expression of miR-206, mTOR, MEF2, MyoD did not change significantly (Supplementary Figure 7). The relationship between miR-133a, SRF and statin-induced muscle injury requires further investigation. We also detected other death pathways in skeletal muscle cells, and the data confirmed that under statin stimulation, skeletal muscle cells had increased necroptosis without significant changes in pyroptosis (Supplementary Figure 8).

A recent report suggests that statin-induced myopathy is associated with the inhibition of mitochondrial complex III [36]. Since mitochondrion has a critical role in apoptosis, this inspired our imagination: Statins-induced mitochondrial complex III inhibition may be related to excessive expression of miR-1a. In the future, we will test this hypothesis.

Nevertheless, our study has several shortcomings: i) Since our study animals were 6-8 weeks old and we did not study aged mice. Previous studies have shown that age is an important factor affecting statin side effects [37]. So, we speculated that the protective effect on statin muscle injury exerted by miR-1a inhibition would be different in aged mice. The effect of age on the protective effect of miR-1a inhibitor would be our next research direction. ii) Simvastatin is a lipophilic statin and most of our studies adopted it. We only have a small number of studies on hydrophilic statin (pravastatin) (Supplementary Figure 6). The results showed that pravastatin also significantly increased the expression of miR-1a, and inhibition of miR-1a similarly reduced statin-induced apoptosis in skeletal muscle cells. However, concluding that 'statins cause skeletal muscle toxicity by increasing miR-1a' may still be somewhat powerless.

ApoE-/- and Ldlr-/- mice are the two most frequently used murine models for hyperlipidemia. According to some reports, experiments on these two models may present different results, and additionally different gender may also affect the experimental results. So, in the next step, we will use Ldlr-/- mice to verify if our results are reproducible and will also present the results of experiments in different genders separately.

In summary, this study reveals a novel mechanism of statin to cause skeletal myopathy. As present in Supplementary Figure 9, statin increases miR-1a expression to decrease MAP3K1, resulting in cell apoptosis of skeletal muscle cells. In this way, statin causes skeletal injury in mice. Therefore, the current study will open some new avenues to investigate the roles of miR-1a in statin’s side effects and also provide some insights to drug design for myopathy in that targeting miR-1a to improve the outcomes of medical intervention.

## Supplementary Material

Supplementary Methods

Supplementary Figures

Supplementary Tables
