# Supplementary material for: Excessive expression of miR-1a by statin causes skeletal injury through targeting mitogen-activated protein kinase kinase kinase 1
Source: Aging (Albany NY). 2021 Apr 16;13(8):11470–90. doi: 10.18632/aging.202839 (PMC8109097; doi:10.18632/aging.202839)
Supplement: Supplementary Methods [file aging-13-202839-s001.pdf]

SUPPLEMENTARY METHODS

Reagents

Primary antibodies against Bcl-2 (ab196495, UK), Bax (ab32503, UK), were obtained from Abcam. Primary antibody against cleaved-caspase3 (#9661, USA), pro-caspase3 (#9662, USA), cleaved-caspase7 (#9491, USA), pro-caspase7 (#9492, USA), cleaved-caspase9 (#9509, USA), pro-caspase9 (#9504, USA) was purchased from Cell Signaling Technology. Primary α-tublin antibody was from Proteintech (11224-1-AP, China) and GAPDH antibody was from Servicebio (GB11002, China). Polyclonal antibodies against MAP3K1 was obtained from SANTA CRUZ (sc-449, USA). Dulbecco Modified Eagle Medium (DMEM) was obtained from Hyclone (SH30022.01B, USA). Fetal bovine serum (FBS) was purchased from Thermo Fisher Scientific (10099141C, USA). Reduced serum medium was from Thermo Fisher Scientific (11058021, USA). Horse serum was from Solarbio (S9050, China). Lipofectamine 3000 was obtained from Invitrogen (L3000015, USA). Simvastatin was from TargetMol (T0687, USA). Simvastatin concentrations were expressed as the final molar concentration in the buffer.

Isolation and differentiation of skeletal muscle satellite cells

Gastrocnemius muscle was isolated from 10 days old suckling mice (C57BL/6) and was cut into small pieces. Collagenase II and trypsin were used to digest muscle tissue at 37° C, and the impurities were removed by 200 mesh cell sieve. After centrifugation for 10 minutes at a speed of 1000 rpm, the cells were suspended in DMEM medium containing 10% fetal bovine serum, and then the cell suspension was placed in a culture flask. The culture flask was placed in an incubator containing 5% CO_2 at 37° C. Muscle satellite cells were isolated by using the difference of the adhesion speed with fibroblasts for a total of 30 min X 3 times. Cell suspension was extracted and placed in a culture flask. 1. 2 × 10^6 cells were seeded in each well of 6-well plate. When the cell density reached 80-90%, DMEM medium containing 10% fetal bovine serum was replaced with DMEM medium containing 2% horse serum. The medium was changed every day. After 3 days, the cells began to differentiate. After 5 days, 90% of satellite cells differentiated into mature skeletal muscle cells with the fusion of skeletal muscle cells into myotubes and MHC positive expression (Supplementary Figure 1).

Transfection of miR-1a mimics and inhibitor *in vitro*

After the muscle satellite cells were well differentiated, the culture medium was removed from 6-well cell culture cluster and washed cells three times with PBS. Mix the miR-1a mimics and inhibitor with the serum-free medium and Lipofectamine 3000 according to the protocol of Lipofectamine 3000. The concentration of inhibitor was 37.5 pmol/ml. 250μl of the mixture was added to each well of 6-well. After 4-6 hours, the reduced serum medium was changed to normal medium containing 10% FBS.

Mutagenesis of miR-1a binding sites in MAP3K1 mRNA 3′-UTR

These reporter constructs were generated in two steps. First, a coding-region fragment containing the miR-1a binding site was generated by PCR and cloned into the pMIR luciferase vector (Ambion) using SpeI and MluI cloning sites. Next, site-directed mutagenesis was performed, introducing three mutations into the binding site's seed sequence in MAP3K1 mRNA 3′-UTR. Subsequently, a DNA fragment containing the 3′-UTR miR-1a binding site was generated by PCR and cloned into pMIR vector, this time using MluI and HindIII sites.

Plasmid transfection into HEK293 and reporter assays

The plasmid constructs (MAP3K1-UTR or MT-MAP3K1-UTR) were co-transfected in HEK293 cells with the pCMV β-gal plasmid and 50 nM each of chemically synthesized miR-1a or negative control oligonucleotides (Applied Biosystems) by using lipofectamine 3000 (Invitrogen). Cells were harvested 48 hours after transfection, and luciferase and β-galactosidase activities were measured.

Construction and infection of lentivirus to cells or mice

The lentivirus construction compassing miR-1a was generated using the AdMax (Microbix) and pSilencer™ adeno 1.0-CMV (Ambion) systems according to the manufacturers' recommendations. Viruses were packaged and amplified in HEK293A cells and purified using CsCl banding followed by dialysis against 10 mM Tris-buffered saline with 10% glycerol. Titering was performed on HEK293 cells using the Adeno-X Rapid Titer kit (BD Biosciences Clontech, Palo Alto, CA, USA) according to the manufacturer's instructions. MiR-1a mimics sequence: 5′-3′ UGGAAUGUAAAGAAGUAUGUAU/ 5′-3′ ACAUACUUCUUUACAUUCCAUU. miR-1a inhibitor sequence: 5′-3′ AUACAUACUUCUUUAC

AUUCCA. Cells were infected with lentivirus overnight in antibiotics-free medium supplemented with 2% FBS. The cells were then washed and incubated in fresh medium for an additional 12 hours before experimentation. For infecting mice, lentivirus containing miR-1a inhibitor sequence or control was injected via tail vein under pressure (in 1 ml of PBS over 5-10s). Lentivirus was injected via tail vein in 100 μl of PBS with 7.6 × 10^7 IFUs of loaded lentivirus. The concentration of miR was 10 mg/kg.

RNA quantification

Total RNA was isolated using a TRIzol-based (Invitrogen) RNA isolation protocol. RNA was quantified by Nanodrop (Agilent Technologies), and RNA and miRNA quality were verified using an Agilent 2100 Bioanalyzer (Agilent Technologies). Samples required 260/280 ratios of more than 1.8, and sample RNA integrity numbers of more than 9 for inclusion. RNA was reverse transcribed using the TaqMan microRNA Reverse Transcription Kit (Applied Biosystems) according to the manufacturer's instructions. Reactions were run for 40 cycles at conditions according to the manufacturer's protocol. U6 was used as a control gene and the target values were normalized to U6 mRNA. miR-1a primer sequences: 5′-GCCGAATGGAATGTAAAGAAGT-3′/5′-TATGGT TTTGACGACTGTGTGAT-3′. MAP3K1 primer sequences: 5′-GTGGTGAAGCCAATCCCTATTA-3′/5′-CTGTCTCCTCCAATCAGGAAAG-3′. U6 primer sequences: 5′-CAGCACATATACTAAAAT TGGAACG-3′/5′-ACGAATTTGCGTGTCATCC-3′. The fold change was calculated using the 2-DD computed tomography method.

Western blotting

Cells or tissues were homogenized on ice in cell-lysis buffer (20 mM Tris-HCl, pH 7.5, 150 mM NaCl, 1 mM Na_2EDTA, 1 mM EGTA, 1% Triton, 2.5 mM sodium pyrophosphate, 1 mM beta-glycerophosphate, 1 mM Na_3VO_4, 1 μg/ml leupeptin) and 1 mM PMSF. Cell was lysated with cell-lysis buffer. The protein content was assayed by BCA protein assay reagent (Pierce, USA). 20 μg proteins were loaded to SDS-PAGE and then transferred to membrane. Membrane was incubated with a 1:1000 dilution of primary antibody, followed by a 1:2000 dilution of horseradish peroxidase- conjugated secondary antibody. Protein bands were visualized by ECL (GE Healthcare). The intensity (area X density) of the individual bands on Western blots was measured by densitometry (model GS-700, Imaging Densitometer; Bio-Rad). The background was subtracted from the calculated area. We used control as 100%.

Immunohistochemistry

The tissue was fixed in 4% paraformaldehyde overnight, and then processed, embedded in paraffin, and sectioned at 4 μm. The deparaffinized, rehydrated section (5 μm) were microwaved in citrate buffer for antigen retrieval. Sections were incubated in endogenous peroxidase (DAKO) and protein block buffer, and then with primary antibodies indicated overnight at 4° C. Slides were rinsed with washing buffer and incubated with labelled polymer-horseradish peroxidase-antimouse/antirabbit antibodies followed by DAB^+ chromogen detection (DAKO). After final washes, sections were counterstained with hematoxylin. All positive staining was confirmed by ensuring that no staining occurred under the same conditions with the use of non-immune rabbit or mouse control IgG.

TUNEL assay

When the cell differentiation on the glass sheet was completed, the glass sheet was removed from the culture medium and fixed in 4% formaldehyde solution for 30 minutes after washing with PBS once. Then, the glass sheets were placed in PBS containing 0.3% Triton X-100 and incubated at room temperature for 5 minutes. TUNEL detection solution according to the instructions of Fluorescent TUNEL Apoptosis Detection Kit (Beyotime, C1088, China) was prepared and mixed well. 50μl of TUNEL detection solution was added to the sample and incubated for 60 minutes in the dark at 37° C. The sheets were sealed with anti-fluorescence quenching solution and observed under fluorescence microscope (Olympus, Japan). The excitation wavelength used ranged from 450 to 500 nm and the emission wavelength ranged from 515 to 565 nm (green fluorescence).

Flow cytometry

Cells were exfoliated with EDTA-free trypsin and transferred to specific tubes for flow cytometry. The cells were treated and dyed according to the procedure recommended by Annexin V PE Apoptosis kit (BD, 559763, USA). The processed samples were kept away from light and detected by flow cytometer (BD, FACS ARIA2, USA) within 1 hour.

Cell counting kit-8 (CCK8) assay

Cells were seeded in 96-well plates and differentiated into mature skeletal muscle cells. Skeletal muscle cells were stimulated according to pre-designed conditions, and appropriate amount of CCK8 (BOSTER, AR1160, China) solution was added to 96-well plates, incubated in a cell

incubator containing 5% CO_2 at 37° C for 1 hour. The absorbance was measured at 450 nm wavelength.

Detection of serum myotoxicity markers

Serum creatine kinase (CK) and lactate dehydrogenase (LDH) in mice were detected as indicators of myotoxicity. Creatine Kinase Assay Kit (Nan Jing Jian Cheng Bioengineering Institute, A032-1-1, China) and Lactate Dehydrogenase Assay Kit (Nan Jing Jian Cheng Bioengineering Institute, A020-1-2, China) was used for the detection. Absorbance was measured at 660 nm and 440 nm wavelength, respectively.

Hanging grid test

The hanging grid test was performed at the end of experiment. Mice were individually placed at the center of a wiremesh screen (2 mm wire thickness). The screen was held 50 cm above a pad. The grid was inverted upside down with the head declining first. The duration of hanging was recorded in three independent trials conducted at least 20 min apart. The data of all three trials were averaged.

Forelimb grip strength test

At the end of experiment, a forelimb grip strength test was performed using a grip strength metre (Handpi HP-5N, China). Mice were held by the tail and grasped a grid with fore paws. The mice were gently pulled by the tail until they released their grip. The forces from three trials were recorded and averaged.

Running tolerance test

Running tolerance test was performed at the end of experiment. The running speed was started at 13 m/min and increased 2 m/min every 3 min. The slop of track was started at 0° C, increased 2° C every 3 min, and maintained at 14° C because of limit of the treadmill. No warm-up was provided before the assessment of running tolerance. The time and distance were recorded when the mice were exhausted.

Hematoxylin eosin stain

Gastrocnemius was fixed in 4% paraformaldehyde for 24 h. Subsequently, the fixed muscles were dehydrated through an ethanol series, treated with xylenes, embedded in paraffin sections, and cut into 5 μm sections. Dewaxing was performed through xylene and ethanol series to deionized water. Stain the nuclei with haematoxylin for 3 min and differentiate with acid ethanol for 30 s. Rinse in the running water until blue up. Then the slides were stained with eosin for 2 min. Dehydrate the slides through ethanol, clear in xylene, and mount. The imaging was performed using Olympus DP72 digital imaging system (Olympus Corporation, Tokyo, Japan). Cross-sectional area (CSA) of muscle fibres was measured using Image Pro Plus. The analysis of CSA was conducted by single investigator blinded to sample.

Statistical analysis

All quantitative results are expressed as mean ± s.e.m. A one-way ANOVA followed by Tukey *post-hoc* tests was used to determine *P* value. Statistical analysis was conducted using IBM SPSS statistics 20.0 (IBM Corp., Armonk, NY, USA) and $P<0.05$ were considered as statistical significance.
