# Supplementary material for: Excessive expression of miR-1a by statin causes skeletal injury through targeting mitogen-activated protein kinase kinase kinase 1
Source: Aging (Albany NY). 2021 Apr 16;13(8):11470–90. doi: 10.18632/aging.202839 (PMC8109097; doi:10.18632/aging.202839)
Supplement: Supplementary Figures [file aging-13-202839-s002.pdf]

SUPPLEMENTARY FIGURES

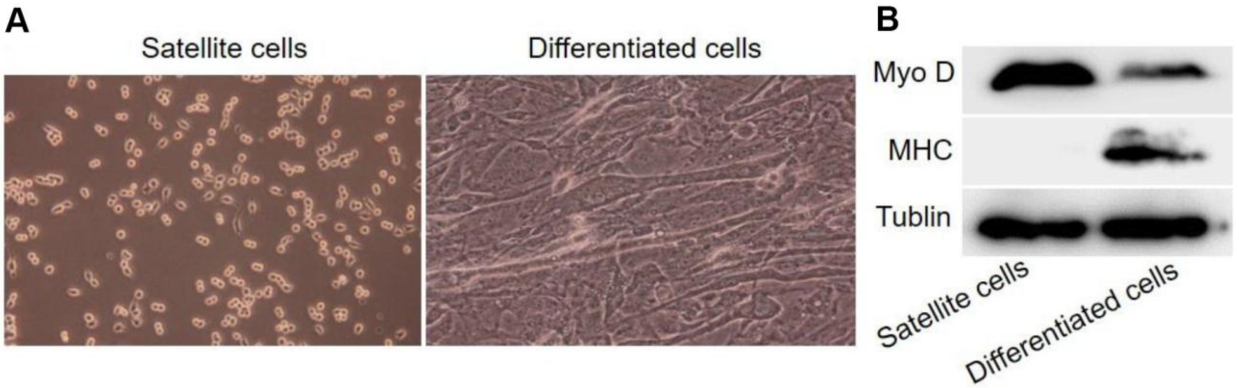

Supplementary Figure 1. Differentiation of skeletal muscle satellite cells to skeletal muscle cells. (**A**) Primary skeletal muscle satellite cells and differentiated skeletal muscle satellite cells with the formation of myotubes. (**B**) Skeletal muscle cells were identified using western blot. MyoD is a biomarker of skeletal muscle satellite cells. MHC is a biomarker of skeletal muscle cells.

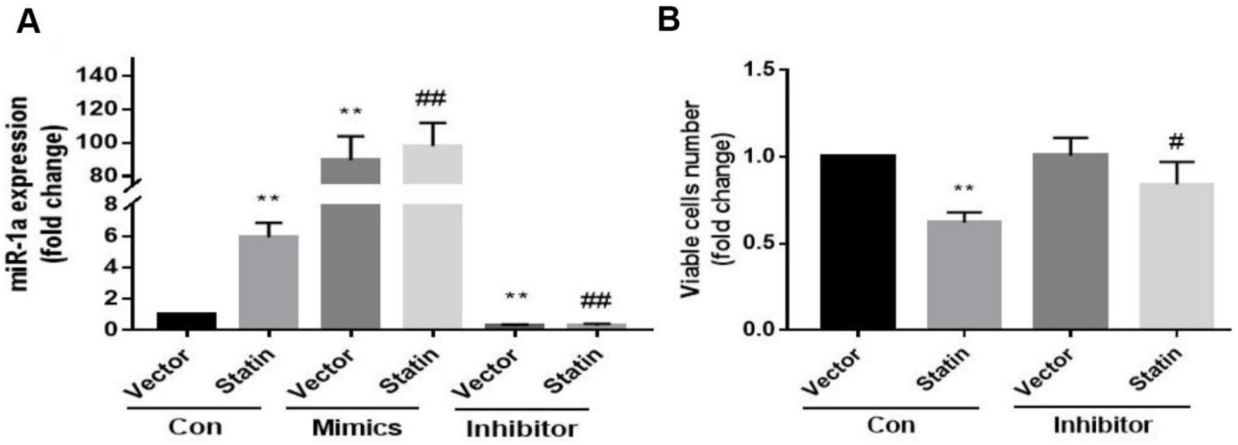

Supplementary Figure 2. Downregulation of miR-1a increases cell viability in skeletal muscle cells treated with statin. (**A**) Cultured skeletal muscle cells were transfected with miR-1a mimics and inhibitor for 48 hours followed by statin treatment for 24 hours. Cells were subjected to detect the levels of miR-1a using real-time PCR. N is 5 in each group. **P<0.01 vs. vector alone. ##P<0.01 vs. statin alone. (**B**) Cultured skeletal muscle cells were transfected with miR-1a inhibitor for 48 hours followed by statin treatment for 24 hours. Cell viability was determined by CCK8 method. N is 5 in each group. **P<0.01 vs. vector alone. #P<0.05 vs. statin alone. A one-way ANOVA followed by Tukey *post-hoc* tests was used to determine *P* value in (**A**, **B**).

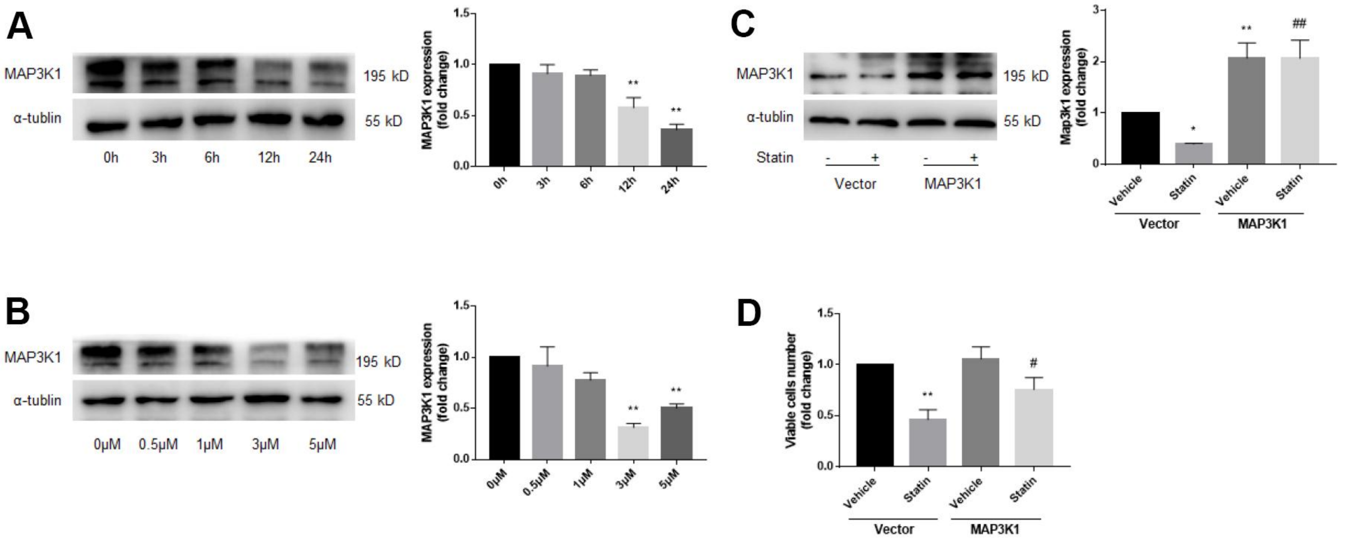

Supplementary Figure 3. Statin reduces the expression of MAP3K1 protein in skeletal muscle cells and MAP3K1 overexpression increases the number of viable cells under statin stimulation. (**A**) Cultured skeletal muscle cells were treated with simvastatin (5 μM) as indicated time points. The expressions of MAP3K1 protein was assayed using western blot. N = 5 per group. $^{**}P < 0.01$ vs. control group (0 h). (**B**) Cultured skeletal muscle cells were treated with simvastatin (24 hours) as indicated concentration points. The expressions of MAP3K1 protein was assayed using western blot. N = 5 per group. $^{**}P < 0.01$ vs. control group (0 μM). (**C**, **D**) Cultured skeletal muscle cells were transfected with plasmids MAP3K1 cDNA for 48 hours followed by statin treatment for 24 hours. Cells were subjected to detect the protein levels of MAP3K1 by western blot in (**C**). Viable cell numbers were determined by the CCK8 method in (**D**) N = 5 per group. $^{**}P < 0.01$ vs. vector alone. $^{\#}P < 0.05$ or $^{\#\#}P < 0.01$ vs. statin alone. A one-way ANOVA followed by Tukey *post-hoc* tests was used to determine *P* value in (**A**–**D**).

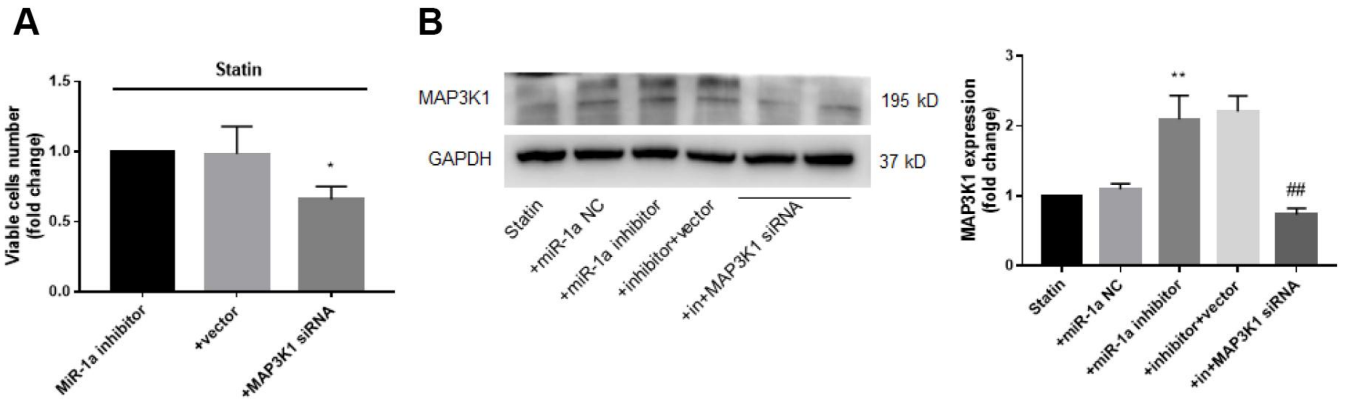

Supplementary Figure 4. The miR-1a-MAP3K1 pathway mediates statin-induced reduction in viable skeletal muscle cell number. Cultured skeletal muscle cells were transfected with miR-1a inhibitor and/or MAP3K1 siRNA for 48 hours followed by statin treatment for 24 hours. (**A**) Viable cell numbers were determined by the CCK8 method. N = 5 per group. $^{*}P < 0.05$ vs. statin+miR-1a inhibitor. (**B**) Cells were subjected to detect the protein levels of MAP3K1. N = 5 per group. $^{**}P < 0.01$ vs. statin alone. $^{\#\#}P < 0.01$ vs. statin+miR-1a inhibitor. A one-way ANOVA followed by Tukey *post-hoc* tests was used to determine *P* value in **A** and **B**.

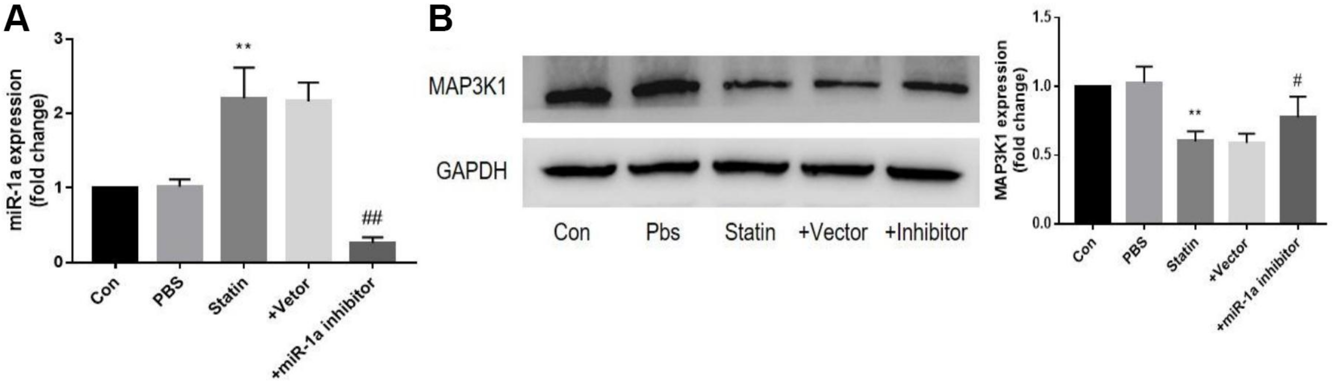

Supplementary Figure 5. Statin decreases the expression of MAP3K1 protein through miR-1a *in vivo*. The protocol for animal experiments was shown in Figure 6A. (**A**) The levels of miR-1a were assayed using real-time PCR. (**B**) The expressions of MAP3K1 protein was assayed using western blot. N is 10-15 in each group. $^{**}P$ < 0.01 vs. PBS group. $^{\#}P$<0.05 or $^{\#\#}P$<0.01 vs. statin alone. A one-way ANOVA followed by Tukey *post-hoc* tests was used to determine *P* value in (**A**, **B**).

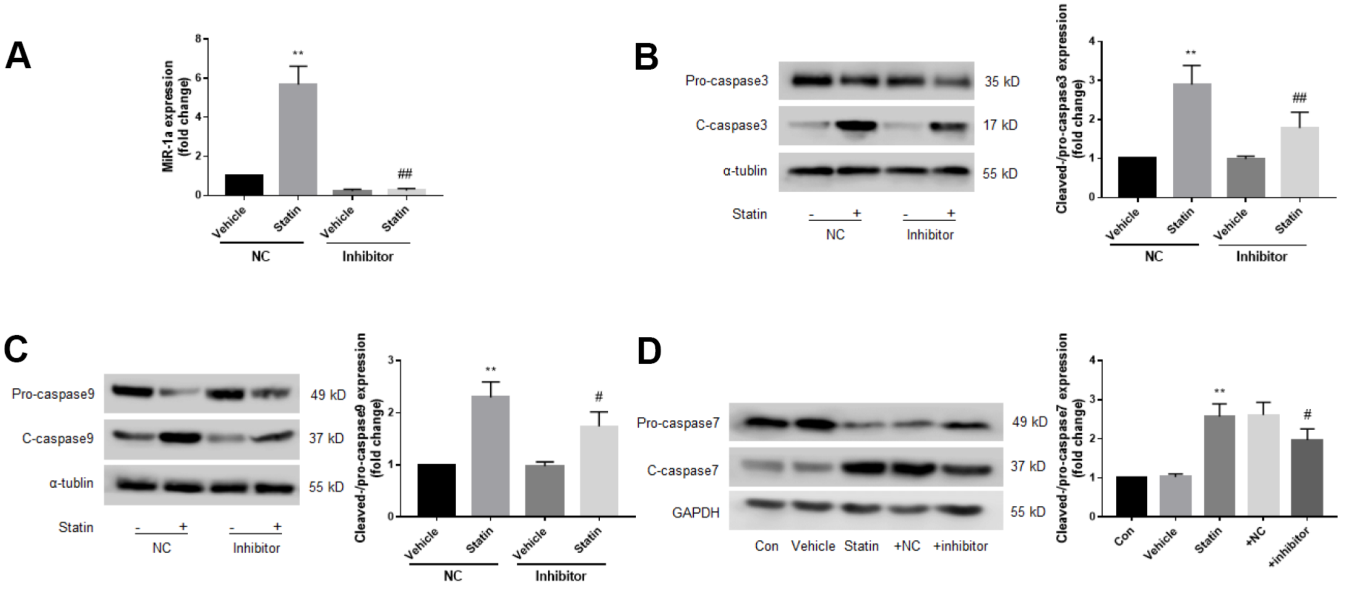

Supplementary Figure 6. Pravastatin (hydrophilic) increases the miR-1a expression in skeletal muscle cells, and inhibition of miR-1a ameliorates statin-induced cell apoptosis. Cultured skeletal muscle cells were transfected with miR-1a inhibitors for 48 hours followed by statin treatment for 24 hours. (**A**) The expression of miR-1a was determined by PCR. (**B**–**D**) Cells were subjected to detect the protein levels of pro-/cleaved-caspase3 in (**B**) pro-/cleaved-caspase9 in (**C**) and pro-/cleaved-caspase7 in (**D**) by western blot. N is 5 in each group. $^{**}P$<0.01 vs. NC alone. $^{\#}P$<0.05 or $^{\#\#}P$<0.01 vs. statin alone. A one-way ANOVA followed by Tukey *post-hoc* tests was used to determine *P* value in (**A**–**D**).

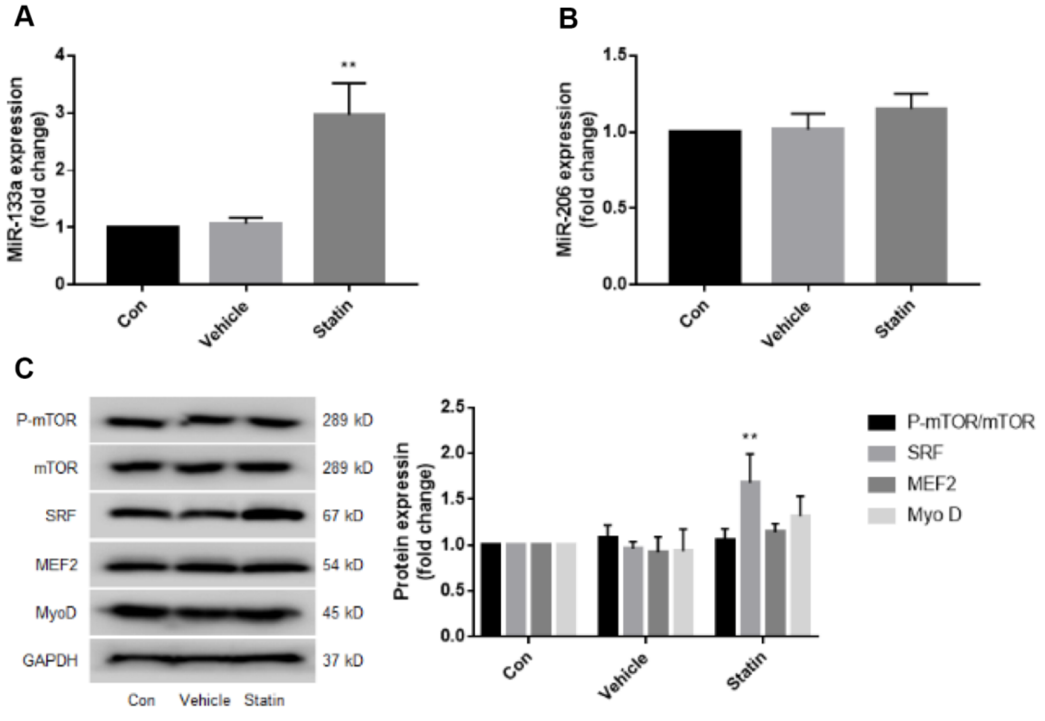

Supplementary Figure 7. After simvastatin stimulation, the expression of miR-133a, SRF increased, while the expression of miR-206, mTOR, MEF2, MyoD did not change significantly. Cultured skeletal muscle cells were stimulated by statin for 24 hours. (**A**, **B**) Cells were subjected to detect the levels of miR-133a in (**A**) and miR-206 in (**B**) by PCR. N is 5 in each group. $^{**}P<0.01$ vs. vehicle alone. (**C**) Cells were subjected to detect the protein levels of mTOR, SRF, MEF2, MyoD by western blot. N is 5 in each group. $^{**}P<0.01$ vs. vehicle alone. A one-way ANOVA followed by Tukey *post-hoc* tests was used to determine *P* value in (**A**–**C**).

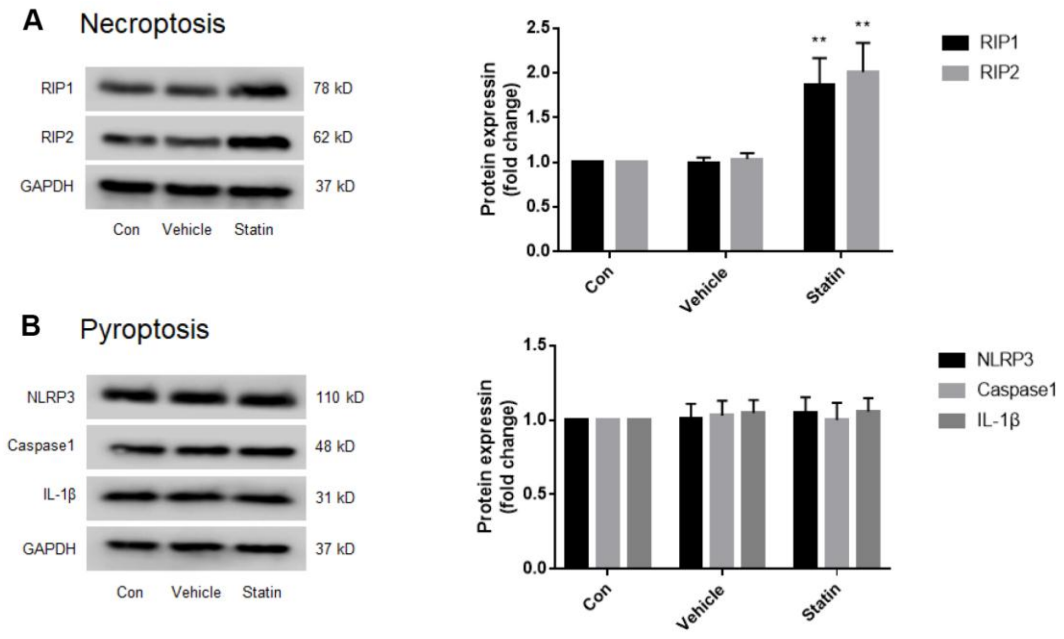

Supplementary Figure 8. Under simvastatin stimulation, the level of necroptosis was significantly increased, while the level of pyroptosis was not. Cultured skeletal muscle cells were stimulated by statin for 24 hours. Cells were subjected to detect the protein levels of RIP1 and RIP2 in (**A**) and NLPP3, caspase1 and IL-1β in (**B**) by western blot. N is 5 in each group. $^{**}P<0.01$ vs. vehicle alone. A one-way ANOVA followed by Tukey *post-hoc* tests was used to determine *P* value in (**A**, **B**).

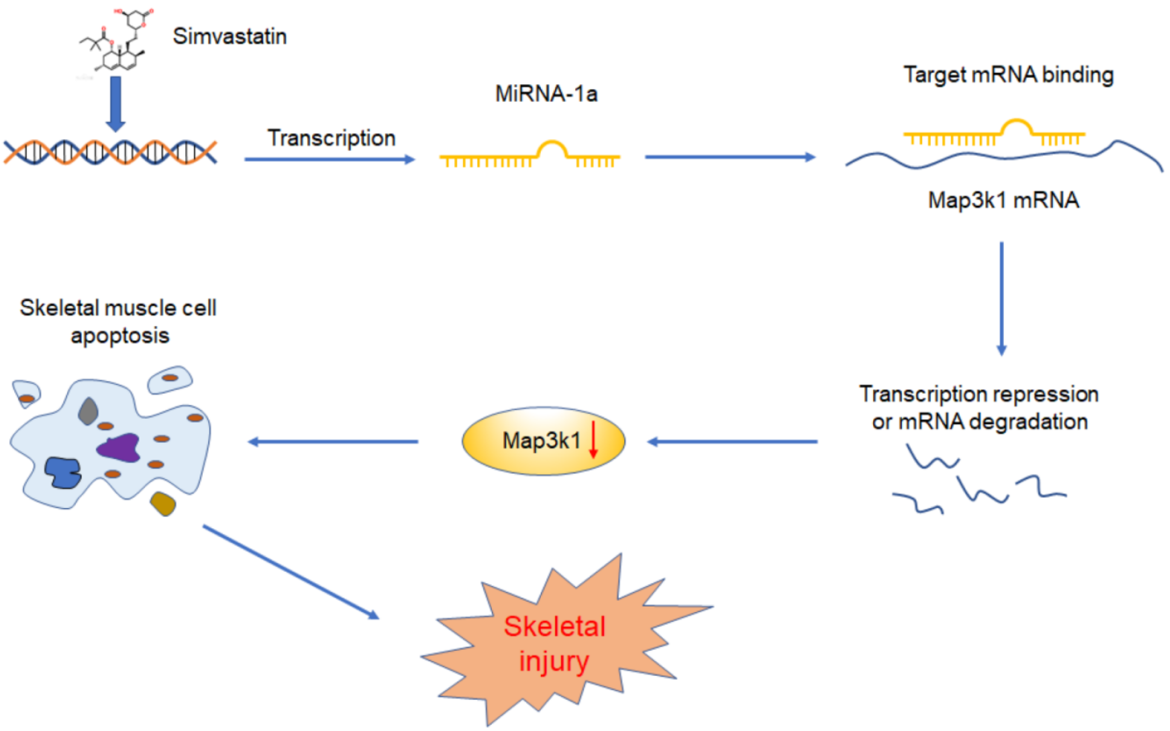

Supplementary Figure 9. Proposed mechanism of statin inducing skeletal myopathy. Statin therapy induces miR-1a excessively expressed in skeletal cells. MiR-1a inhibits MAP3K1 gene expression by targeting 3'-UTR of MAP3K1 mRNA, resulting in apoptosis of skeletal muscle cells. In this way, statin causes skeletal myopathy.
