# Supplementary material for: Excessive expression of miR-1a by statin causes skeletal injury through targeting mitogen-activated protein kinase kinase kinase 1
Source: Aging (Albany NY). 2021 Apr 16;13(8):11470–90. doi: 10.18632/aging.202839 (PMC8109097; doi:10.18632/aging.202839)
Supplement: Supplementary Tables [file aging-13-202839-s003.pdf]

SUPPLEMENTARY TABLES

Supplementary Table 1. Body weight of mice in each group (g).

Group	-2 w	-1 w	0 w	1 w	2 w	3 w	4 w	5 w	6 w	7 w	8 w
Con	16.3±3.4	16.9±2.8	17.8±3.0	19.4±3.2	21.8±2.9	22.4±3.5	23.9±4.1	24.6±3.7	26.4±4.6	27.8±4.3	29.6±4.5
Vehicle	16.1±2.9	17.2±3.1	17.6±3.2	18.9±2.9	20.5±3.4	22.3±3.3	24.2±3.9	24.7±3.9	25.9±4.2	26.7±4.2	28.4±4.7
Statin	16.2±3.1	16.8±2.7	17.5±2.7	18.2±2.7	18.4±2.5	19.7±2.3	20.2±2.5*	21.7±3.0	22.5±3.1	22.8±3.3*	23.3±3.2*
+NC	16.5±2.2	16.9±2.9	17.8±2.5	18.5±3.3	18.7±2.9	19.5±2.8	20.5±2.3	21.3±2.9	21.8±3.2	22.3±3.0	23.1±3.1
+inhibitor	16.2±3.1	17.0±2.8	17.6±2.9	18.1±2.8	18.6±2.3	20.3±2.2	21.7±2.1	22.9±2.2	24.4±3.0	25.2±3.1	26.9±3.5

*P<0.05 vs. vehicle group.

Supplementary Table 2. Food intake of mice in each group (g/day/mouse).

Group	-2 w	-1 w	0 w	1 w	2 w	3 w	4 w	5 w	6 w	7 w	8 w
Con	3.1±0.4	3.7±0.5	4.2±0.6	4.8±0.5	5.2±0.6	5.4±0.7	5.8±0.7	6.3±0.8	6.8±1.1	7.3±0.8	7.5±1.2
Vehicle	3.2±0.6	3.6±0.8	4.1±0.5	5.0±0.7	5.3±0.6	5.5±0.6	5.7±0.8	6.2±0.7	6.5±0.9	7.1±1.0	7.4±0.9
Statin	3.1±0.5	3.5±0.6	4.1±0.6	4.9±0.7	5.1±0.5	5.2±0.7	5.2±0.6	5.7±0.5	5.9±0.6	6.0±0.6**	6.2±0.8**
+NC	3.0±0.4	3.6±0.6	3.9±0.5	4.8±0.6	5.2±0.7	5.1±0.5	5.3±0.7	5.6±0.6	5.7±0.5	6.2±0.5	6.1±0.7
+inhibitor	3.1±0.7	3.5±0.7	4.0±0.4	4.7±0.5	5.3±0.6	5.4±0.6	5.4±0.5	6.0±0.7	6.1±0.8	6.6±0.9	6.9±0.7

**P<0.01 vs. vehicle group.

Supplementary Table 3. Serum total cholesterol concentrations of mice in each group (mmol/L).

Group	0w	8w
Con	14.5±2.4	15.3±2.8
Vehicle	14.2±3.1	14.7±2.9
Statin	15.6±2.6	10.4±1.2 **
+NC	14.3±2.4	10.9±1.7
+inhibitor	14.8±2.9	11.5±1.3

**P<0.01 vs. vehicle group.
